# Recovery of Sentence Production Processes Following Language Treatment in Aphasia: Evidence from Eyetracking

**DOI:** 10.3389/fnhum.2017.00101

**Published:** 2017-03-13

**Authors:** Jennifer E. Mack, Michaela Nerantzini, Cynthia K. Thompson

**Affiliations:** ^1^Department of Communication Sciences and Disorders, Northwestern UniversityEvanston, IL, USA; ^2^Cognitive Neurology and Alzheimer's Disease Center, Northwestern UniversityEvanston, IL, USA; ^3^Department of Neurology, Northwestern UniversityEvanston, IL, USA

**Keywords:** aphasia, sentence production, eyetracking, online processing, agrammatism, language treatment

## Abstract

**Introduction:** Sentence production impairments in aphasia often improve with treatment. However, little is known about how cognitive processes supporting sentence production, such as sentence planning, are impacted by treatment.

**Methods:** The present study used eyetracking to examine changes in sentence production resulting from a 12-week language treatment program focused on passive sentences (Treatment of Underlying Forms (TUF); Thompson and Shapiro, 2005). In two pre-treatment and two post-treatment sessions, nine participants with mild-to-moderate agrammatic aphasia performed a structural priming task, which involved repeating primed sentences (actives or passives) and then, using the same verb, producing sentences describing pictured events. Two individuals with aphasia performed the eyetracking task on the same schedule without intervening language treatment. Ten unimpaired older adults also performed the task to identify normal performance patterns. Sentence production accuracy and speech onset latencies were examined, and eye movements to the pictured Agent and Theme characters were analyzed in the first 400 ms after picture onset, reflecting early sentence planning, and in the regions preceding the production of the sentence subject and post-verbal noun, reflecting lexical encoding.

**Results:** Unimpaired controls performed with high accuracy. Their early eye movements (first 400 ms) indicated equal fixations to the Agent and Theme, consistent with structural sentence planning (i.e., initial construction of an abstract structural frame). Subsequent eye movements occurring prior to speech onset were consistent with encoding of the correct sentence subject (i.e., the Agent in actives, Theme in passives), with encoding of the post-verbal noun beginning at speech onset. In participants with aphasia, accuracy improved significantly with treatment, and post-treatment (but not pre-treatment) eye movements were qualitatively similar to those of unimpaired controls, indicating correct encoding of the Agent and Theme nouns for both active and passive sentences. Analysis of early eye movements also showed a treatment-induced increase in structural planning. No changes in sentence production accuracy or eye movements were found in the aphasic participants who did not receive treatment.

**Conclusion:** These findings indicate that treatment improves sentence production and results in the emergence of normal-like cognitive processes associated with successful sentence production, including structural planning.

## Introduction

One of the defining features of agrammatic aphasia is impaired production of grammatical sentences, which affects the ability to communicate effectively and efficiently. In spontaneous speech, speakers with agrammatism often produce short and syntactically simple utterances, with frequent errors of grammatical morphology and thematic mapping, i.e., mapping between verb-argument structure and grammatical functions such as subject and object (Saffran et al., 1989; Bird and Franklin, 1995; Thompson et al., 1995, 2013a; Rochon et al., 2000). Sentence production also is impaired in structured language tasks (Caplan and Hanna, 1998; Faroqi-Shah and Thompson, 2003; Bastiaanse and Edwards, 2004; Thompson and Lee, 2009; Thompson et al., 2013a). Production of complex syntactic forms, including noncanonical structures such as passive sentences (e.g., *The man was lifted by the woman*), is generally more impaired than production of syntactically simple forms such as active sentences (e.g., *The woman was lifting the man*) (Faroqi-Shah and Thompson, 2003; Burchert et al., 2008; Thompson and Lee, 2009; Thompson et al., 2013a). Accounts of these production deficits have centered on damaged or incomplete syntactic representations (Friedmann and Grodzinsky, 1997), impaired grammatical processing (Burchert et al., 2008; Cho and Thompson, 2010; Lee et al., 2015) or deficits in more general processing resources (Kolk, 1995).

Two major treatment approaches for impaired complex sentence production have been developed: Treatment of Underlying Forms (TUF; Thompson and Shapiro, 2005) and Mapping Therapy (Schwartz et al., 1994). Both approaches aim to strengthen the ability to encode a thematic representation (i.e., “who did what to whom”) as a grammatical sentence structure. This involves training the linguistic properties of verbs in terms of their verb-argument and thematic role properties in both simple (e.g., active) and syntactically complex sentences (e.g., passive structures). Whereas Mapping Therapy does this through relatively implicit forms of training (e.g., picture/sentence associations), TUF explicitly trains sentence-building processes, guided by the properties of the verb (see review in Faroqi-Shah and Thompson, 2012). The rationale for this feature of TUF is that training the processes that underlie typical sentence production should result in more normal-like sentence production in individuals with agrammatism. In addition, TUF and Mapping Therapy differ with respect to the role of grammatical complexity. Whereas Mapping Therapy protocols typically proceed from simple to complex structures (e.g., Schwartz et al., 1994), in TUF, complex structures are trained in order to promote generalization to related structures of lesser complexity, for example, from complex to simpler syntactic structures (see reviews in Thompson et al., 2003; Thompson and Shapiro, 2005) and from complex to simpler verb-argument structures (Thompson et al., 2013b). In a meta-analysis, TUF was found to lead to robust treatment and generalization effects in people with mild-to-moderate agrammatism (Dickey and Yoo, 2010).

Relatively little is known, however, about how (or if) the sentence production system changes in response to TUF. In the domain of sentence comprehension, Dickey and Thompson (2004) found that agrammatic listeners who received TUF, compared to those who did not, were better at detecting syntactic anomalies in noncanonical sentences, suggesting that TUF may lead to more normal-like online sentence comprehension. Mack and Thompson (in press) also found that treatment-induced improvement in offline sentence comprehension resulted in more normal-like eye movements (i.e., agent-first looking patterns in correct responses) in an online sentence-picture matching task in 10 individuals with chronic agrammatic aphasia. Notably, in another study (Mack et al., 2016), we found consistent eyetracking patterns over intervals with no treatment (e.g., high test-retest reliability) in individuals with aphasia. This latter finding, coupled with the results of Mack and Thompson (in press) suggests that treatment normalizes online sentence comprehension strategies. However, we are aware of no previous studies that have examined online sentence production strategies used by aphasic speakers prior to or after treatment.

Several studies have addressed processes engaged by unimpaired speakers during sentence production using eyetracking. For example, studies have recorded eye movements while participants produce sentences describing two-character (Agent, Theme) scenes (e.g., a mailman chasing a dog) (Griffin and Bock, 2000; Gleitman et al., 2007; Cho and Thompson, 2010; Kuchinsky and Bock, 2010; Hwang and Kaiser, 2014; Konopka and Meyer, 2014; Van de Velde et al., 2014). Other studies have monitored eye movements as participants construct sentences with arrays of words (Lee and Thompson, 2011a,b). Results show that speakers tend to fixate a depicted character, or written word, prior to producing it. These studies also have elucidated two ways in which sentence production is planned by unimpaired speakers, depending on linguistic demands (Ferreira and Swets, 2002; Kuchinsky and Bock, 2010; Wagner et al., 2010; Konopka and Meyer, 2014; Van de Velde et al., 2014; see review in Bock and Ferreira, 2014). When speakers use *structural planning*, they first generate an abstract structural frame of the sentence (sometimes guided by the argument structure of the verb), and then retrieve words in the sentence. For example, when describing a picture of a mailman chasing a dog, the first 300-400 ms are used to identify the event (e.g., *chase*) and plan a corresponding abstract structural frame (e.g., a transitive frame). During this time participants direct fixations equally to the Agent and Theme, after which preferential fixation to the character to be expressed as the sentential subject occurs (Griffin and Bock, 2000; also see Bock et al., 2003, 2004). In contrast, when speakers use *word-by-word planning*, visual attention to one character (e.g., *mailman*) within 200 ms of picture onset precipitates its selection as the grammatical subject of the to-be-produced sentence (Gleitman et al., 2007).

Research focused on agrammatic sentence planning suggests that both modes of planning are used, but structural planning is used more often compared to unimpaired speakers, particularly when linguistic demands are relatively high (e.g., in production of sentences containing verbs with complex argument structures; Lee et al., 2015). However, it does not always result in successful sentence production (Saffran and Martin, 1997; Hartsuiker and Kolk, 1998; Marin and Schwartz, 1998; Cho and Thompson, 2010; Verreyt et al., 2013; Cho-Reyes et al., 2016). For example, Cho and Thompson (2010) found that although aphasic speakers show successful structural priming, reflecting structural planning, primed sentences contain a high rate of role-reversal errors, indicating faulty thematic mapping.

The present study used eyetracking to examine the effects of training passive sentences on aphasic speakers' sentence production planning strategies.^1^ Before and after language treatment, participants' eye movements were tracked as they produced active and passive sentences (using a structural priming task, following Cho and Thompson, 2010). The aims of this study were as follows. First, we tested whether TUF resulted in improved production of passive sentences, which we expected based on the results of previous studies (Thompson et al., 1997; Jacobs and Thompson, 2000). Second, we tested whether TUF resulted in more normal-like production processes, as reflected by speech onset latencies and online eye movements. We also administered two eyetracking sessions during baseline and on follow-up testing to examine reliability of eye movements. Our expectation was that TUF would result in more normal-like eye movements, reflecting improvements in sentence planning, but not necessarily more rapid sentence production. This is because TUF trains the processes hypothesized to underlie normal sentence production, but does not emphasize processing speed. We expected that eye movement changes would reflect improved structural sentence planning, given that TUF emphasizes verb-based sentence building and structural priming paradigms promote structural planning (Van de Velde et al., 2014). Finally, we expected stable eye movement patterns across sessions as shown in a previous study (Mack et al., 2016).

## Method

### Participants

The study participants included 10 unimpaired older adults without aphasia, all native English speakers who reported normal or corrected-to-normal vision and hearing and no history of speech, language, or learning disorders. Eleven individuals with aphasia participated in the study: nine received language treatment (*Treatment group*) and two did not (*Natural History group*).^2^ The older adult controls were marginally older than the participants with aphasia [Aphasia M (SD) = 48 (12); Control M (SD) = 58 (11); *p* = 0.051; two-tailed *t-*test], but the two groups did not differ with respect to years of education [Aphasia M (SD) = 16.6 (2.2); Control M (SD) = 17.4 (2.5); *p* = 0.47; two-tailed *t-*test]. The two individuals in the Natural History group also did not differ from the Treatment group with respect to age and education (Crawford-Howell two-tailed *t*-tests, *p*'s > 0.05). The inclusion criteria for the participants with aphasia was as follows: (1) left-hemisphere stroke at least one year prior to the study; (2) pass vision and hearing screenings (a pure-tone audiometric screening at 40 dB, 1,000 Hz); (3) no pre-stroke history of speech and language impairments; (4) safe to participate in magnetic resonance imaging (a component of the study not reported here); (5) mild-to-moderate aphasia with agrammatic features, including impaired production and comprehension of passive sentences (see details below); (6) preserved single-word comprehension and at most moderate motor speech deficits. Of 26 participants screened for this study between 2013 and 2015, 11 were enrolled.^3^ The study was approved by the Institutional Review Board at Northwestern University and all participants provided informed consent.

All participants with aphasia (see demographic and language scores in Table 1) exhibited mild-to-moderate agrammatism. Aphasia Quotients (AQs) from the Western Aphasia Battery-Revised (WAB-R) (Kertesz, 2006), reflecting overall aphasia severity, ranged from mild to moderately severe (range: 53.5–89). The Treatment and Natural History participants did not differ with respect to WAB-R AQ (Crawford-Howell two-tailed *t*-test, *p*'s > 0.05). All evinced relatively unimpaired noun production, as illustrated by scores ≥75% correct on the Confrontation Naming subtest of the *Northwestern Naming Battery* (NNB) (Thompson and Weintraub, 2014) and verb production was more impaired than noun production at the group level, with two participants showing verb naming scores below 75% (A01, A04). Single-word comprehension, of both nouns and verbs, was relatively preserved in all participants, as indicated by scores ≥80% on the Auditory Comprehension subtest of the NNB. Crucially, all participants showed greater difficulty producing noncanonical, as compared to canonical, sentences, as indicated by performance on the Sentence Production Priming Test (SPPT) of the *Northwestern Assessment of Verbs and Sentences* (NAVS) (Thompson, 2011): noncanonical range: 0–53.3% correct; canonical range: 33.3–100% correct. Unimpaired speakers have been shown to perform at ceiling on this task across sentence types (M's > 98%) (Cho-Reyes and Thompson, 2012). Impaired sentence comprehension was also evident in all participants, with 8 of 11 (all except A01, A02, and NH02) showing better comprehension of canonical than noncanonical sentences (Sentence Comprehension Test from the NAVS). Narrative language samples (Cinderella story) were also collected and analyzed using the Northwestern Narrative Language Analysis System (Thompson et al., 2012). Reduced speech rate (words per minute) (i.e., greater than two standard deviations below the mean of 13 unimpaired older adult controls), indicating nonfluent speech, was evident in all participants except one (A06) (older adult control *M* = 132.2, *SD* = 18.8; Thompson et al., 2012). In addition, all participants except one (A02) exhibited impaired grammatical production, as measured by a reduced proportion of grammatical sentences compared to healthy older adult controls (older adult control *M* = 93.0%, *SD* = 4.4%).

**Table 1:** **Demographic information and language testing scores for individuals with aphasia**.

**ID**	**Age**	**Gender**	**Education (Years)**	**Months Post-onset**	**WAB-R AQ**	**NNB (CN) Nouns (%)**	**NNB (CN) Verbs (%)**	**NNB (AC) Nouns (%)**	**NNB (AC) Verbs (%)**	**NAVS SPPT C (%)**	**NAVS SPPT NC (%)**	**NAVS SCT C (%)**	**NAVS SCT NC (%)**	**WPM**	**% GS**
A01	51	M	16	82	69.7	87.5	62.5	100.0	100.0	66.7	0.0	66.7	66.7	42.3	9.1
A02	35	F	19	58	83.7	100.0	93.8	100.0	100.0	80.0	46.7	66.7	73.3	54.4	93.3
A03	52	F	16	73	75.8	87.5	93.8	96.7	100.0	73.3	46.7	86.7	53.3	42.8	64.7
A04	53	F	13	104	53.5	75.0	50.0	96.7	93.3	80.0	46.7	93.3	33.3	36.4	0.0
A05	53	M	21	39	74.1	100.0	75.0	100.0	100.0	60.0	0.0	80.0	26.7	32.2	6.7
A06	41	M	16	16	89	100.0	100.0	100.0	100.0	80.0	33.3	86.7	66.7	120.0	78.1
A07	48	M	16	17	85	93.8	100.0	83.3	86.7	66.7	13.3	80.0	40.0	49.7	45.5
A08	22	F	14	31	77.7	81.3	87.5	93.3	100.0	100.0	53.3	93.3	66.7	46.1	70.6
A09	64	M	18	19	75.6	100.0	93.8	100.0	100.0	46.7	26.7	86.7	40.0	72.2	46.7
NH01	41	M	16	85	76.2	100.0	93.8	100.0	100.0	80.0	33.3	86.7	46.7	26.0	46.7
NH02	64	M	18	13	71.1	93.8	81.3	96.7	100.0	33.3	0	46.7	46.7	64.0	64.3
Mean	48		16.6	48.8	75.6	92.6	84.7	97.0	98.2	69.7	27.3	79.4	50.9	53.3	47.8
SD	12		2.2	32.8	9.4	8.8	16.1	5.0	4.3	18.2	20.8	14.1	15.6	25.9	30.9

*WAB-R AQ, Western Aphasia Battery-Revised, Aphasia Quotient; NNB, Northwestern Naming Battery; CN, Confrontation Naming subtest; AC, Auditory Comprehension subtest; NAVS, Northwestern Assessment of Verbs and Sentences; SPPT, Sentence Production Priming Test; SCT, Sentence Comprehension Test; C, Canonical Sentences; NC, Noncanonical sentences; WPM, Words Per Minute; % GS, % Grammatical sentences. Participants A01-A09 were in the Treatment group; Participants NH01-02 were in the Natural History group. NH02 entered the treatment group (A09) following participation in the Natural History group*.

### Treatment method (TUF)

Using principles and methods of Treatment of Underlying Forms (TUF; Thompson and Shapiro, 2005), participants received training for 12 weeks^4^, in twice-weekly sessions of approximately 90 min each. Following pre-treatment administration of sentence production probes in the baseline phase, consisting of passive sentences (*n* = 60) and other sentence structures (*n* = 60)^5^, participants were trained to produce long passive sentences with locative adjuncts (*n* = 10 training sentences, e.g., *The boy was shaved by the man at the barbershop*). During the production training phase, participants were presented with an action picture (e.g., a boy being shaved by a man at a barbershop) and word cards corresponding to the active form of the training sentence (e.g., *The man was shaving the boy at the barbershop*), and were asked to identify the thematic roles of pictured elements [“action” (verb), “doer” (Agent, e.g., *man*), “receiver” (Theme, e.g., *boy*), and “location”]. Then, the experimenter guided the participant in building an active sentence with word cards. Following the principles of TUF (i.e., training the abstract linguistic generalizations that relate syntactically complex to simple forms), the experimenter trained participants to build a passive sentence from the active sentence, emphasizing differences between passive and active sentences with respect to morphosyntax [i.e., changes in verb from present-participle (*shaving*) to past-participle (*shaved*)] and thematic mapping (i.e., Theme displacement to the subject position, Agent placement in post-verbal adjunct position, with addition of *by*). The participant then practiced building the passive sentence independently using word cards. Participants received an equal amount of comprehension training, which consisted of identification of thematic roles as well as experimenter-led sentence building. Feedback from the experimenter was provided at each step. In the post-treatment phase, sentence production was again tested using the same items as in the pre-treatment probes.

Participants in the Treatment group also performed four control tasks before and after treatment^6^. These tasks, selected from the Psycholinguistic Assessments of Language Processing in Aphasia (PALPA; Kay et al., 1992), assessed aspects of language that were unrelated to the treatment protocol: minimal pair discrimination (PALPA subtest 1); oral reading (PALPA subtest 35), spelling to dictation (PALPA subtest 40), and word-semantic associations (PALPA subtest 51). These tasks were expected to elicit stable performance from pre- to post-treatment.

### Eyetracking task

The eyetracking task was based on Cho and Thompson (2010). Participants repeated prime sentences (e.g., *The gorilla was lifted by the chimp*) and then were asked to use the primed verb and structure to describe a picture containing different characters (e.g., *The man was lifted by the woman*). Our main variables of interest were accuracy, latency, and online eye movements in the picture description component of the task, although we also examined prime repetition accuracy. This task was designed to reduce demands on verb retrieval and morphosyntactic encoding during picture description, both of which are impaired in agrammatic aphasia (see reviews in Bastiaanse and Thompson, 2012; Druks, 2017). However, the picture description task required mapping between thematic roles and syntactic structures, which was the primary process of interest in this study. For example, in passive trials, participants were required to identify the Theme and Agent in the picture and map them, respectively, to the subject and post-verbal adjunct positions.

### Stimuli

We selected 28 semantically-reversible transitive verbs with regular past participles, none of which were included in the training materials. For each verb, we constructed one active and one passive prime sentence (e.g., *The chimp was lifting the gorilla/The gorilla was lifted by the chimp)*, for a total of 56 experimental trials. Each prime pair shared the same noun phrases, which were gender-neutral referring to humans [e.g., *student, reporter, kid, artist; M* ratings between 3 and 5 on the gender-bias norms of (Kennison and Trofe, 2003); (1–7 scale)] in 21 sentence pairs and to animals (e.g., *gorilla, dog, cat, donkey*) for the other 7. In half of the prime pairs, the identity of the Agent was switched between active and passive trials (e.g., *The chimp was lifting the gorilla/The chimp was lifted by the gorilla*). The length (in syllables), log frequency (Corpus of Contemporary American English; Davies, 2008), and degree of gender-neutrality of the two noun phrases did not differ between the active and passive conditions (*p*'s > 0.05). The prime sentences were recorded by a male native English speaker at a slightly slower than normal speech rate (*M*: 3.8 syllables/second), to facilitate participants' ability to understand and repeat the sentences. Table 2 summarizes the properties of the prime sentences.

**Table 2:** **Means (standard deviations) of properties of prime sentence stimuli, by experimental condition**.

	**Active**	**Passive**
N1 Length (syllables)	2.21 (0.83)	2.46 (0.88)
N1 Log Frequency	3.79 (0.71)	3.84 (0.62)
N1 Gender Norms (males)	4.04 (0.35)	4.02 (0.34)
N1 Gender Norms (females)	3.96 (0.26)	3.94 (0.30)
N2 Length (syllables)	2.36 (0.87)	2.11 (0.79)
N2 Log Frequency	3.76 (0.62)	3.71 (0.70)
N2 Gender Norms (males)	4.02 (0.34)	4.02 (0.31)
N2 Gender Norms (females)	3.95 (0.26)	3.98 (0.22)
Speech Rate (syllables/second)	3.85 (0.26)	3.77 (0.27)

*N1, the first noun of the sentence (grammatical subject); N2, the second (post-verbal) noun*.

**Figure 1:**
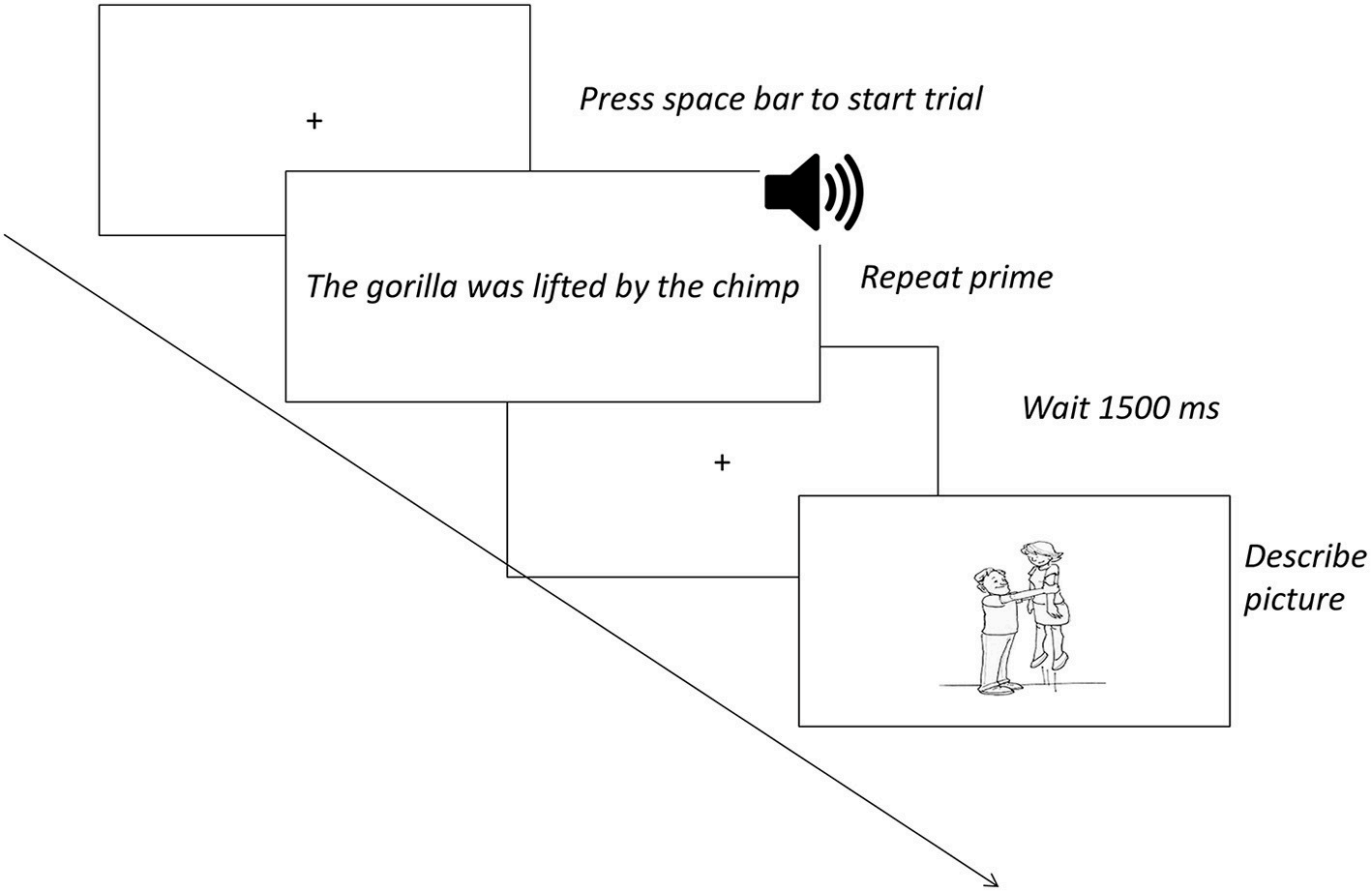
**Example stimulus for a passive sentence trial**. The italic text outside the boxes indicates what participants did in each step of the trial.

Each verb was depicted in a line drawing with two human characters (one male, one female), semantically unrelated to the nouns in prime sentences, spatially separated and of equal size and visual complexity (see Figure 1; e.g., a man lifting a woman). To keep lexical processing demands low, all pictures could be felicitously described using the nouns *man, woman, boy*, and/or *girl*. For half of the verbs, the identity of the pictured Agent was switched between active and passive trials. Within each condition, the Agent was equally likely to appear in the left and right portions of the scene, and the male and female characters were equally likely to be Agents. The trials were presented in a pseudorandom order, with no more than three active or passive trials in a row. Active/passive pairs containing the same verb appeared at least 9 trials apart. There were no filler trials.

### Procedure

Participants were seated in front of a 16″ × 10″ computer monitor in a dimly lit room, with visual stimuli centered on the computer screen. The experimental stimuli were presented using Superlab (Cedrus). Participants' eye movements were monitored using an Applied Science Laboratory (ASL) model D6/Eye-Trac 6,000 remote eye tracker with a sampling rate of 60 Hz and precision of approximately 0.5° of visual angle. The eyetracker was calibrated at the beginning of the test session and, as needed, every eight trials thereafter.

Participants were given the following instructions: “In this experiment, you will be repeating sentences and making sentences that go with pictures. First, you will hear a sentence and repeat it. Then, you will see a picture of the same action. Make a sentence like the one you just said.” Two trials (one active, one passive) were modeled for the participant. Then, the participant completed four practice trials (two active, two passive). Each practice trial included feedback about the target response (e.g., “For this picture, a good sentence is The boy was followed by the girl”). These instructions and feedback were intended to promote the use of the primed structures. Experimental trials (see Figure 1) began with a fixation cross. Then, the participant pressed the space bar to trigger auditory and visual presentation of the prime sentence, which remained on the screen as the participant repeated it. The participant then pressed the space bar again, triggering a 1,500 ms cross followed by the target picture stimulus, which the participant described. Five hundred millisecond prior to onset of the target picture, a beep was presented to time-lock participants' spoken responses to the onset of the picture stimulus. The participant pressed the space bar to trigger each trial.

Unimpaired controls performed the task once, in a single test session lasting approximately 30 min. Participants with aphasia performed the task four times: twice upon entry into the study (*M* = 4.4 days apart; *SD* = 4.3)^7^ and twice at study end (*M* = 3.9 days apart, *SD* = 4.4), after completion of the 12-week TUF program in the Treatment group and a 12-week no-treatment period in the Natural History group.

### Data analysis

#### Accuracy and error type coding

Analyses of accuracy and error type were performed by at least two researchers, with disagreements resolved by consensus. For prime repetition, responses were considered correct if they contained all of the following: (1) the primed verb morphology (actives: auxiliary verb + present participle, e.g., *was lifting*; passives: auxiliary verb + past participle, e.g., *was lifted*) or a grammatical and unambiguous substitution (e.g., actives: *lifted/lifts/is going to lift*, passives: *was being lifted*); (2) the target verb or a transitive semantically-related substitute verb (e.g., *carry* for *lift*); (3) two noun phrases, one pre-verbal and one post-verbal; (4) *by* before the second noun phrase in passives, but no preposition before the second noun phrase in actives. Phonological paraphasias (sharing at least 50% of phonemes of the target word) were accepted, as were omitted determiners (e.g., *gorilla* for *the gorilla*) and inserted words when they did not change the target sentence structure (e.g., *little boy* for *boy*). The specific grammatical form and lexical content of the noun phrases was not critical for the priming manipulation, and thus substitution of one or both nouns was accepted. When participants produced multiple attempts to repeat the prime, we coded the most accurate attempt.

For the picture description responses, we followed the criteria (1–4) as described above. However, coding of picture description responses differed in two key ways from that of the prime responses. First, because the lexical content of the nouns was critical for assessing thematic mapping, we only accepted noun substitutions under certain circumstances. Second, we coded only participants' initial utterance for each trial. In order to identify the initial utterance, we divided the sentence into three regions: *N1*, which started with the first attempt to produce the subject noun; *V*, which started with the first attempt to produce the main verb; and *N2*, which started with the first attempt to produce the second noun and ended with the final attempt to produce that noun. Reformulations were allowed within a sentence region (e.g., within N1: *The boy …no, the woman*); when these occurred, we coded the final attempt within that region. However, reformulations to earlier sentence regions (e.g., *The boy chased …No, the woman chased*) were not considered part of the initial utterance and were not coded. Errors in picture description were classified into six mutually exclusive categories (see Table 3).

**Table 3:** **Error coding scheme**.

**Error type**	**Definition**	**Example of error (correct sentence: ***The woman was lifted by the man***)**
Correct Unprimed Structure	Unprimed structure, but grammatically and thematically correct (i.e., active for passive or vice versa)	*The man was lifting the woman*
Role Reversal	Reversal of Agent and Theme	*The man was lifted by the woman*
Morphosyntactic	Error(s) in verbal morphology; omission, addition, or substitution of *by*	*The woman was lift by the man The woman was lifted the man*
Noun Substitution (not otherwise classified as Role Reversal)	Substitution of one or both nouns; same-gender or gender-neutral substitutions (e.g., *boy* or *cashier* for *man*) were not counted as errors, when the identity of the referent was thematically correct	*The man was lifted by the man*
Verb Substitution	Substitution of the main verb; semantically-appropriate, transitive substitutions were not counted as errors (e.g., *carry* for *lift*)	*The woman was chased by the man*
Incomplete/No Response (NR)/Multiple Errors	Incomplete utterance (omitted N1, V, and/or N2); no response; two or more of the above error types	*The woman was … The man was lift the woman*

#### Speech coding

The temporal boundaries of each sentence region (N1, V, N2) were marked in Praat (Boersma and Weenink, 2016) by two researchers, with any disagreements resolved by consensus. Speech onset latencies (i.e., the time prior to production of N1) were entered into statistical analyses.

#### Eye movement data: preprocessing

Eye movement fixations were tallied using EYENAL (Applied Science Laboratories) and coded as either Agent or Theme fixations. These were pre-determined areas of interest on target pictures (i.e., rectangles surrounding the Agent and Theme; subtending approximately 15° of visual angle vertically and 11° of visual angle horizontally). A fixation was defined as a gaze of at least 100 ms in duration, within one degree of visual angle. For visualization, the data were aggregated into 100 ms bins time-locked to the onset of the picture. We computed the total time that each participant spent fixating the Agent and Theme characters for each trial and sentence region (Onset, PreN1, N1, V, N2). As input to statistical analyses, these data were binarized: a greater amount of time spent fixating the Agent resulted in an *Agent advantage*, whereas a greater amount of time spent fixating the Theme reflected a *Theme advantage*. The Onset region spanned the first 400 ms after picture onset; this window was selected based on previous work indicating that early fixations (within 400 ms) can be indicative of sentence planning strategies (Griffin and Bock, 2000; Konopka and Meyer, 2014). The PreN1 window spanned from 400 ms after picture onset to N1 onset, whereas the N1, V, and N2 regions were the same as described above. Data points were excluded if the total fixation time in a region was less than 100 ms, indicating insufficient data for computing the Agent/Theme advantage.

#### Statistical models

Mixed-effects logistic regression was used to test for changes in passive sentence production accuracy (i.e., effects of study Phase) in the pre- vs. post-treatment probe data for the Treatment group, but not for the Natural History group (due to the small number of participants). As for all mixed-effects logistic regression models, we computed *z*-values and *p*-values (*lme4* package in R; Bates et al., 2015). All models contained simple-coded predictor variables, and initially contained maximal random effects structures (i.e., random intercepts and random slopes on Participant and Item for all fixed factors). If the model did not initially converge, the random effects for Item were reduced until convergence took place^8^. The degrees of freedom and final random effects structures were computed for each model. Performance on the control tasks (PALPA 1, 35, 40, and 51) was compared from pre- to post-treatment using paired two-tailed *t*-tests.

Accuracy data for prime repetition and picture description in the structural priming (eyetracking) task were also analyzed using mixed-effects logistic regression. For unimpaired controls, we tested for effects of Sentence Type (active prime, passive prime) on accuracy. For participants with aphasia in the Treatment group, we tested for effects of Sentence Type, Phase (pre-treatment, post-treatment), and their interaction. In the presence of a significant interaction (*p* < 0.05), we computed simple effects of Phase (i.e., treatment effects) for each sentence type, and simple effects of Sentence Type (i.e., active vs. passive primes) for each phase^9^. In separate models for the participants with aphasia, we tested for stability in performance between the two pre-treatment sessions and the two post-treatment sessions by testing for effects of Sentence Type, Session, and their interaction. Again, because of the small number of participants in the Natural History group, we did not perform statistical analyses on these data.

Further analyses were conducted to test for treatment-related changes in the types of errors produced by participants with aphasia in the picture description task. For this analysis, error data were collapsed across the two sessions obtained at pre- and post-treatment. For each error type that was produced in at least 5% of trials, changes in *absolute error rate* (i.e., the proportion of all trials with errors) were examined using mixed-effects logistic regression, with the same model specification procedures as described above for overall accuracy. Given that multiple error types were analyzed, a multiple comparisons correction (False Discovery Rate (FDR); Benjamini and Hochberg, 1995) was used to correct *p*-values for the predictors of interest^10^.

Analyses of speech onset latencies were performed using mixed-effects linear regression (reporting *t*-values and *p*-values computed with the lme4 and lmerTest packages in R, Bates et al., 2015; Kuznetsova et al., 2015). Speech onset latency data were transformed and outliers were removed^11^, resulting in normally-distributed variables in each data set (Shapiro-Wilk test, *p*'s > 0.1). For unimpaired controls, we tested for effects of Sentence Type (active vs. passive prime) on speech onset latencies. The analysis was restricted to correct trials in unimpaired controls; due to high accuracy in this group, incorrect trials were rare and presumably not representative of typical sentence production processes. However, due to the high error rate in the Treatment group, we performed separate sets of analyses, one combining all trials (correct and incorrect) and one with correct responses only. In the analysis of all trials, we tested for effects of Sentence Type, Phase, and their interaction. Importantly, the Sentence Type variable was determined by the structure of the prime, rather than what participants produced. We also tested for any effects of Session (i.e., session 1 vs. session 2) within the pre-treatment and post-treatment phases. Second, we tested for effects of Sentence Type in the correct trials for the pre- and post-treatment data. Only participants who produced at least five correct actives and five correct passives in a study phase were included in the analyses of that phase. Five participants (A02, A06, A07, A08, and A09) produced five or more correct active and passive sentences pre-treatment, and thus were included in the pre-treatment analyses, whereas eight participants (A01-08) produced at least five correct sentences of each structure post-treatment, and were included in those analyses. Due to relatively sparse data, we did not examine effects of Session in the analyses of the correct trials.

Mixed-effects logistic regression was used to analyze the eye movement data (i.e., the Agent/Theme advantage), modeled separately for each sentence region (Onset, PreN1, N1, V, N2). As for the speech onset latency data, separate analyses were conducted for the data from all trials and the data from correct trials only, and effects of Session were examined. Due to the large number of models used to analyze the eye data, the *p-*values for predictors of interest were corrected for multiple comparisons, using the FDR method^12^.

#### Individual performance patterns: treatment effects and comparison with natural history group

For participants with aphasia, we examined individual patterns of performance for three measures of passive sentence production: pre- to post-treatment change (or pre- to post-study change for Natural History participants) in prime repetition accuracy, picture description accuracy, and the proportion of trials with a Theme advantage (i.e., majority of time spent fixating the Theme) in the PreN1 region. This eye movement measure was chosen because it reflects the use of thematic structure to encode the sentence subject (i.e., eye movements to the Agent in actives and the Theme in passives before speech onset). Within the Treatment group, we tested whether these measures were correlated (Pearson *r*, two-tailed, FDR-corrected for multiple comparisons) to delineate patterns of recovery. In order to determine whether behavioral changes from pre- to post-treatment in the Treatment group were attributable to TUF (vs. practice effects), the change in performance patterns over time in the Natural History participants were compared to that of the Treatment group (Crawford-Howell *t*-tests, one-tailed).

## Results

### Treatment (TUF) results

The Treatment group showed improved accuracy on sentence production probes for passive sentences from the pre-treatment phase (M: 11.7%, SD: 13.9%) to the post-treatment phase (M: 80.9%, SD: 13.1%) (*z* = 9.847, *p* <0.001). On an individual level as well, all participants showed increased accuracy following treatment. The final model of the data included random intercepts for Participant and Item and random by-participant slopes for Phase (df (6, 1074)). In contrast to the improvement seen in the Treatment group, the Natural History participants showed stable performance on the probe task [M (SD) at study entry: 10.0% (14.1%); M (SD) at study end: 11.7% (14.1%)].

### Control tasks

Table 4 summarizes the pre- and post-treatment performance of the Treatment group on the control tasks (PALPA 1, 35, 40, and 51). Paired *t*-tests revealed no significant changes on any measure (*p*'s > 0.05).

**Table 4:** **Accuracy (% correct) for control tasks in the Treatment group**.

	**Pre-treatment**		**Post-treatment**	
	***M* (%)**	***SD* (%)**	***M* (%)**	***SD* (%)**
PALPA 1 Minimal pair discrimination	92.3	7.0	93.6	5.0
PALPA 35 Oral reading	78.5	21.8	77.1	20.2
PALPA 40 Spelling to dictation	35.9	28.6	37.8	31.2
PALPA 51 Word-semantic associations	65.8	11.8	72.9	15.5

### Eyetracking task: prime repetition

The prime repetition results appear in Table 5 (summary statistics) and 6 (statistical models). The unimpaired controls performed at ceiling on prime repetition (>99% correct) and thus these data were not analyzed statistically. For the Treatment group, accuracy improved significantly from the pre-treatment phase to the post-treatment phase (main effect of Phase, *z* = 2.306, *p* < 0.05), with no significant effects of Sentence Type or interaction between Phase and Sentence Type (*p'*s > 0.05). Performance was stable across the two pre-treatment test sessions and the two post-treatment sessions, as indicated by the absence of any significant main effects of Session or interactions between Session and Sentence Type (*p*'s > 0.05). The Natural History participants showed consistent prime repetition accuracy between study entry and study end (change of no more than 0.04 in the proportion of correct responses).

**Table 5:** **Accuracy (proportion of correct trials) for prime repetition and picture description**.

	**Unimpaired adults**		**Treatment group Pre-treatment**		**Treatment group Post-treatment**		**Natural history group Study entry**		**Natural history group Study end**	
	***M***	***SD***	***M***	***SD***	***M***	***SD***	***M***	***SD***	***M***	***SD***
**PRIME REPETITION**										
Active	1.00	0.00	0.85	0.16	0.86	0.17	0.86	0.05	0.90	0.04
Passive	1.00	0.01	0.70	0.36	0.84	0.20	0.80	0.18	0.76	0.32
**PICTURE DESCRIPTION**										
Active	0.95	0.05	0.40	0.19	0.49	0.27	0.18	0.23	0.13	0.18
Passive	0.92	0.15	0.14	0.18	0.63	0.18	0.05	0.08	0.04	0.03

### Eyetracking task: picture description

#### Accuracy

Summary statistics for picture description accuracy appear in Table 5; statistical model results appear in Table 6. The unimpaired controls performed with high accuracy (*M* = 93% overall) with no significant differences between actives and passives (*p* > 0.05). The Treatment group showed significantly improved accuracy from pre- to post-treatment (main effect of Phase, *z* = 4.173, *p* < 0.001), with no effect of Sentence Type (*p* > 0.05). However, a significant interaction between Phase and Sentence Type was observed (*z* = 3.293, *p* = 0.001). Simple effects analyses indicated that treated aphasic speakers produced active sentences more accurately than passives pre-treatment (*z* = −2.501, *p* < 0.05) but there was no difference in accuracy between the two structures at post-treatment (*p* > 0.05), indicating significant improvement for passive sentences (*z* = 4.881, *p* < 0.001) but not actives (*p* > 0.05). The models of performance within the pre-treatment phase and post-treatment phase revealed no significant main effects or interactions with Session (*p*'s > 0.05). The Natural History participants showed consistent performance from study entry to study end (change of no more than 0.05 in the proportion of correct responses).

**Table 6:** **Mixed-effects logistic regression models: prime repetition and picture description accuracy**.

	**Prime repetition**		**Picture description**	
**Unimpaired adults**	*Not analyzed: At ceiling*		*Df* (8, 552)	
			*z*	*p*
Intercept^P, I^			6.462	<0.001
Sentence Type^P, I^			0.851	0.395
**Treatment group**	*Df* (24, 1936)		*Df* (24, 1936)	
	*z*	*p*	*z*	*p*
Intercept^P, I^	3.824	<0.001	−2.966	0.003
Phase^P, I^	2.306	0.021	4.173	<0.001
Sentence Type^P, I^	−1.483	0.138	−1.125	0.260
Phase ^*^ Sentence Type^P, I^	1.729	0.084	3.293	0.001
Simple effect of Phase: Actives			0.687	0.492
Simple effect of Phase: Passives			4.881	<0.001
Simple effect of Sentence Type: Pre-treatment			−2.501	0.012
Simple effect of Sentence Type: Post-treatment			1.387	0.166

*Reference levels are as follows: Sentence Type, active; Phase, pre-treatment. Significant effects (p<0.05), other than effects on the intercept, are shaded. Simple effects are reported only in the presence of a significant interaction term. Superscripts indicate random effects that were included in the final model: P, Participant; I, Item. Df, degrees of freedom*.

#### Error types

The rate of errors by type in picture description for the Treatment and Natural History groups is summarized in Table 7; results of statistical models appear in Table 8. For the treatment group there was an interaction between Phase and Sentence Type for Correct Unprimed Structure errors (*z* = −3.541, *p* < 0.01), which increased from pre- to post-treatment for actives (*z* = 2.006, *p* < 0.05) but decreased for passives (*z* = −3.363, *p* = 0.001). The rate of Incomplete/NR/Multiple Type errors decreased overall with treatment (*z* = −3.980, *p* < 0.001), and there was also an interaction between Phase and Structure (*z* = −2.997, *p* < 0.05); simple effects analyses demonstrated that the rate of these errors significantly decreased for passives (*z* = −5.515, *p* < 0.001) but not actives (*p* > 0.05). No other significant effects were found. Error rates remained largely consistent from study entry to study end in the Natural History group. For passive sentences, the largest reduction in error rate from study entry to study end (0.07) was observed for morphosyntactic errors.

**Table 7:** **Error types produced by participants with aphasia: *Absolute error rate* (i.e., proportion of all trials with errors of each type)**.

	**Correct unprimed structure**		**Role reversal**		**Morpho-syntactic**		**Noun substitution**		**Verb substitution**		**Incomplete/NR/Multiple types**	
	***M***	***SD***	***M***	***SD***	***M***	***SD***	***M***	***SD***	***M***	***SD***	***M***	***SD***
**TREATMENT GROUP**												
Active, Pre	0.06	0.13	0.04	0.04	0.14	0.20	0.05	0.04	0.03	0.04	0.28	0.15
Active, Post	0.17	0.22	0.08	0.07	0.07	0.07	0.01	0.02	0.03	0.04	0.15	0.08
Passive, Pre	0.18	0.11	0.15	0.20	0.07	0.08	0.02	0.03	0.00	0.01	0.44	0.20
Passive, Post	0.05	0.06	0.06	0.06	0.09	0.08	0.02	0.03	0.03	0.03	0.13	0.09
**NATURAL HISTORY GROUP**												
Active, Entry	0.01	0.01	0.01	0.01	0.29	0.33	0.00	0.00	0.02	0.00	0.50	0.53
Active, End	0.03	0.04	0.01	0.01	0.39	0.13	0.00	0.00	0.01	0.01	0.44	0.37
Passive, Entry	0.04	0.05	0.33	0.47	0.29	0.39	0.00	0.00	0.02	0.03	0.27	0.03
Passive, End	0.06	0.09	0.31	0.44	0.22	0.29	0.00	0.00	0.00	0.00	0.37	0.27

*Pre, Pre-Treatment; Post, Post-Treatment; Entry, Study Entry; End, Study End; NR, No Response*.

**Table 8:** **Mixed-effects regression models of errors produced by participants with aphasia (Treatment group): *Absolute error rate* (i.e., proportion of all trials with errors of each type)**.

	**Correct unprimed structure**		**Role reversal**		**Morpho-syntactic**		**Incomplete/NR/Multiple types**	
	***Df*****(20,1940)**		***Df*****(24,1936)**		***Df*****(24,1936)**		***Df*****(24,1936)**	
	***z***	***P***	***z***	***p***	***z***	***p***	***z***	***P***
Intercept^P, I^	−7.172	<0.001	−10.57	<0.001	−9.838	<0.001	−5.376	<0.001
Phase^P, I^	−0.313	0.823	0.611	0.740	0.501	0.740	−3.980	0.001
Sentence Type^P, I^	1.017	0.618	−0.117	0.907	−0.558	0.740	1.298	0.473
Phase ^*^ Sentence Type^P(all), I (all except Correct Unprimed Structure)^	−3.541	0.002	−0.791	0.735	1.289	0.473	−2.997	0.011
Simple effect of Phase: Actives	2.006	0.045					−1.371	0.170
Simple effect of Phase: Passives	−3.363	0.001					−5.515	<0.001

*Reference levels are as follows: Sentence Type, active; Phase, pre-treatment. Significant effects (p<0.05), other than effects on the intercept, are shaded. Simple effects are reported only in the presence of a significant interaction term. P-values for main effects and interactions were corrected for multiple comparisons (see text for details). Superscripts indicate random effects that were included in the final model: P, Participant; I, Item. Df, degrees of freedom*.

#### Speech onset latencies

The speech onset latency data for each group are summarized in Table 9. For the unimpaired adults, speech onset latencies did not differ across Sentence Types (*p* > 0.05). For the Treatment group, in the analysis containing all trials (regardless of response accuracy), there were no significant effects of Sentence Type or Phase (*p*'s > 0.05). There were also no effects of Session in the pre-treatment or post-treatment data (*p*'s > 0.05). In the analyses containing correct trials only, there were no significant effects of Sentence Type in either the pre- or post-treatment data (*p*'s > 0.05). Speech onset latencies were also numerically consistent from study entry to study end in the Natural History group (mean change < 0.3 s). An analysis of speech onset latencies in correct trials was not possible for this participant group due to the small sample size and low number of correct trials. Similarly, effects of test session were not investigated due to the size of the data set.

**Table 9:** **Mean (SD) speech onset latencies (seconds)**.

	**Unimpaired adults Correct trials**		**Treatment group All trials Pre-tx**		**Treatment group All trials Post-tx**		**Treatment group Correct trials Pre-tx**		**Treatment group Correct trials Post-tx**		**Natural history group All trials Study entry**		**Natural history group All trials Study end**	
	***M***	***SD***	***M***	***SD***	***M***	***SD***	***M***	***SD***	***M***	***SD***	***M***	***SD***	***M***	***SD***
Active	2.08	0.52	4.25	2.21	5.06	1.58	4.79	1.85	4.18	1.28	5.48	2.60	5.73	3.21
Passive	1.92	0.54	4.42	2.54	5.16	1.57	4.83	1.50	5.44	2.31	5.52	2.14	5.29	2.46

*Pre-Tx, pre-treatment; Post-Tx, post-treatment. The “Active” and “Passive” labels refer to the structure of the prime*.

#### Eye movements

**Figure 2:**
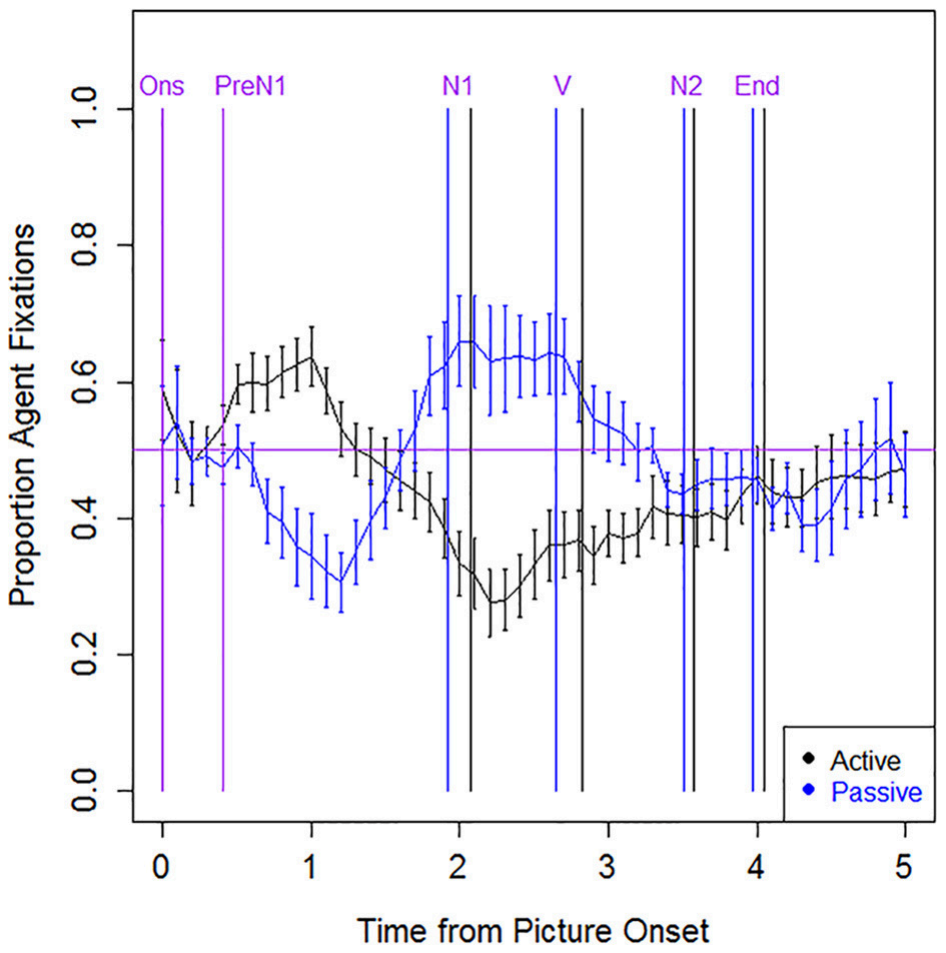
**Eye movement data: Unimpaired speakers, correct trials**. The x axis indicates the time from picture onset in seconds; the y axis indicates the proportion of fixations to the Agent, out of all fixations to the Agent and Theme. Vertical lines indicate the mean onset time for each sentence region (active in black; passive in blue; labels, and onset times that are the same across structures, are in purple). The horizontal line indicates at-chance fixation patterns (i.e., an equal proportion of fixations to the Agent and Theme). Ons, picture onset; PreN1, beginning of PreN1 region (400 ms); N1, onset of subject noun; V, onset of verb; N2, onset of post-verbal noun; End, trial end.

Statistical models of the eye movement data are summarized in Table 10. For unimpaired speakers (Figure 2), fixation patterns did not differ between active and passive sentences in the Onset region (first 400 ms after picture onset; *p* > 0.05). However, they showed more Agent advantage (and fewer Theme advantage) trials in active than in passive sentences during the PreN1 region (from 400 ms after picture onset to the onset of N1) (*z* = −7.241, *p* < 0.001), indicating encoding of the grammatical subject before speech onset. After speech onset (N1 and V regions), the unimpaired speakers had more Agent advantage trials in passive than in active sentences (N1: *z* = 5.854, *p* < 0.001; V: *z* = 8.135, *p* < 0.001), indicating encoding of N2. During the N2 region, fixation patterns did not reliably differ between active and passive sentences (*p* > 0.05).

**Table 10:** **Mixed-effects logistic regression models of eye movement data**.

	**Onset**		**PreN1**		**N1**		**V**		**N2**	
**Unimpaired adults, correct trials**	***Df(5, 316)***		***Df(8, 513)***		***Df(8, 500)***		***Df(8, 506)***		***Df(8, 487)***	
	***z***	***p***	***z***	***p***	***z***	***p***	***z***	***p***	***z***	***p***
Intercept^P(V, N2), I(all)^	0.296	0.767	−1.478	0.139	−0.481	0.630	−1.270	0.204	−1.804	0.071
Sentence Type^P (all except Onset), I (all)^	0.023	0.982	−7.241	<0.001	5.854	<0.001	8.135	<0.001	0.332	0.925
**Treatment group, all trials**	***Df(24, 1075)***		***Df (24, 1914)***		***Df(24, 1861)***		***Df (24,1726)***		***Df(24, 1638)***	
	***z***	***p***	***z***	***p***	***z***	***p***	***z***	***p***	***z***	***p***
Intercept^P, I^	−0.021	0.983	1.045	0.296	−0.454	0.650	−2.144	0.032	−1.709	0.087
Phase^P, I^	0.289	0.837	−2.874	0.009	−1.254	0.262	3.260	0.003	3.157	0.004
Sentence Type^P, I^	0.156	0.876	−3.723	0.001	−1.747	0.110	3.695	0.001	3.691	0.001
Phase ^*^ Sentence Type^P, I^	0.277	0.837	−2.615	0.017	−2.463	0.023	2.116	0.051	3.250	0.003
Simple effect of Phase: Actives			−0.955	0.340	0.125	0.901			1.014	0.310
Simple effect of Phase: Passives			−5.153	<0.001	−2.230	0.026			3.689	<0.001
Simple effect of Sentence Type: Pre-Treatment			−0.926	0.355	0.671	0.502			0.877	0.381
Simple effect of Sentence Type: Post-Treatment			−3.421	0.001	−2.560	0.011			3.903	<0.001
**Treatment group, correct trials (Pre;** ***n*** = **5)**	***Df(5,73)***		***Df(5,150)***		***Df(5,148)***		***Df(3,148)***		***Df(5,135)***	
	***z***	***p***	***z***	***p***	***z***	***p***	***z***	***p***	***z***	***p***
Intercept^P (all except N2)^	−0.222	0.824	0.378	0.705	0.034	0.973	−1.646	0.100	0.038	0.970
Sentence Type^P (all except V)^	−2.541	0.014	−3.718	<0.001	−1.349	0.177	6.540	<0.001	4.149	<0.001
**Treatment group, correct trials (Post;** ***n*** = **8)**	***Df(2,292)***		***Df(5,506)***		***Df(5,503)***		***Df(5,502)***		***Df(5,483)***	
	***z***	***p***	***z***	***p***	***z***	***p***	***z***	***p***	***z***	***p***
Intercept^P (all except Onset)^	0.746	0.456	−1.302	0.193	−0.780	0.436	−0.615	0.538	0.273	0.785
Sentence Type^P (all except Onset)^	−0.856	0.392	−6.760	<0.001	−3.654	<0.001	7.376	<0.001	5.087	<0.001

*Reference levels are as follows: Sentence Type, active; Phase, pre-treatment. Pre, pre-treatment; Post, post-treatment. Significant effects (p < 0.05), other than effects on the intercept, are shaded. Simple effects are reported only in the presence of a significant interaction term. P-values for main effects and interactions were corrected for multiple comparisons (see text for details). Superscripts indicate random effects that were included in the final model. P, Participant; I, Item. Df, degrees of freedom*.

#### Treatment group: all trials

**Figure 3:**
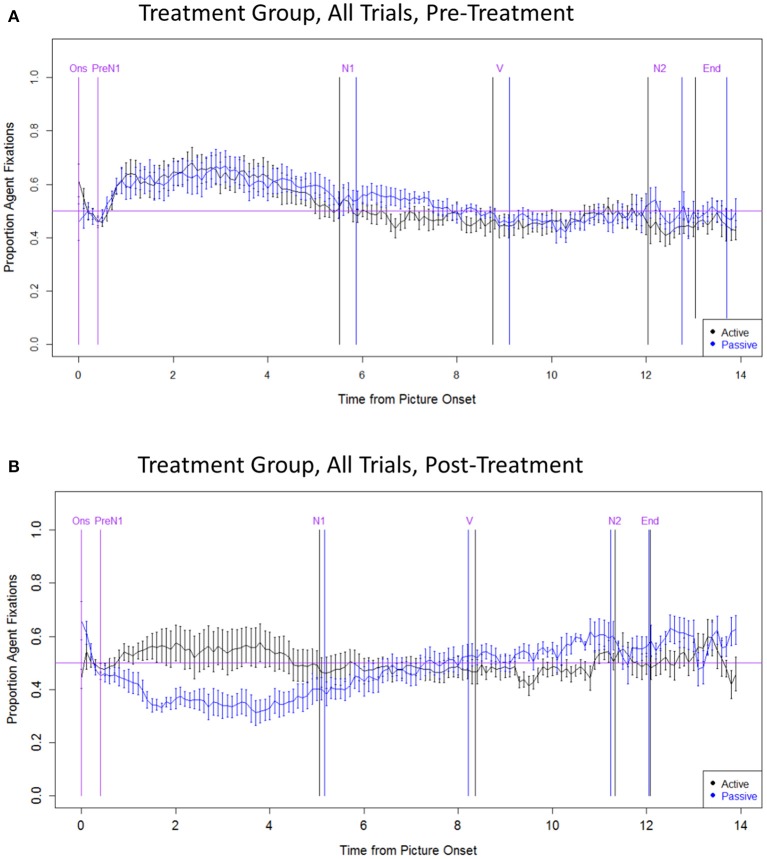
**Eye movement data: Treatment group, all trials**. **(A)** Pre-treatment data, **(B)** Post-treatment data. The x axis indicates the time from picture onset in seconds; the y axis indicates the proportion of fixations to the Agent, out of all fixations to the Agent and Theme. “Active” and “Passive” data points refer to trials with active and passive primes, respectively. Vertical lines indicate the mean onset time for each sentence region (active in black; passive in blue; labels, and onset times that are the same across structures, are in purple). The horizontal line indicates at-chance fixation patterns (i.e., an equal proportion of fixations to the Agent and Theme). Ons, picture onset; PreN1, beginning of PreN1 region (400 ms); N1, onset of subject noun; V, onset of verb; N2, onset of post-verbal noun; End, trial end.

The first set of analyses examined changes in eye movements from pre- to post-treatment, combining all trials (regardless of response accuracy; see Figure 3). No significant effects were observed in the **Onset** region (*p*'s > 0.05). In the **PreN1** region, the speakers in the Treatment group had more Theme advantage (and fewer Agent advantage) trials post-treatment vs. pre-treatment (main effect of Phase: *z* = −2.874, *p* < 0.01), and in passive vs. active sentences (main effect of Sentence Type: *z* = −3.723, *p* = 0.001). Notably, there was a significant interaction between Phase and Sentence Type (*z* = −2.615, *p* < 0.05), such that the rate of Theme advantage trials increased following treatment for passive sentences (*z* = −5.153, *p* < 0.001) but no significant changes were observed for active sentences (*p* > 0.05). Eye movement patterns during PreN1 did not differ between active and passive sentences pre-treatment (*p* > 0.05); however, post-treatment, participants had significantly more Theme advantage trials in passives vs. actives (*z* = −3.421, *p* = 0.001). In the **N1** region, there was a significant interaction between Phase and Sentence Type (*z* = −2.463, *p* < 0.05), such that the rate of Theme advantage trials increased for passives (*t* = −2.230, *p* < 0.05), with no change for actives (*p* > 0.05). The eye movement patterns for the PreN1 and N1 regions suggest more robust encoding of the Theme as the subject of passives post-treatment.

In the **V and N2** regions, participants had more Agent advantage trials for passives than for actives (V: *z* = 3.695, *p* = 0.001; N2: *z* = 3.691; *p* = 0.001), and at post-treatment vs. pre-treatment (V: *t* = 3.260, *p* < 0.01; N2: *t* = 3.157, *p* < 0.01). In the N2 region only, there was a significant interaction between Phase and Sentence Type (*z* = 3.250, *p* < 0.01), driven by an increase in Agent advantage trials for passive sentences (*z* = 3.689, *p* < 0.001), but no change for actives (*p*'s > 0.05). These results indicate more consistent encoding of N2 (the Agent) in passive sentences post-treatment.

Eye movement patterns were found to be stable across test sessions within the pre-treatment data as well as the post-treatment data, as indicated by the absence of any significant main effects or interactions with Session (*p*'s > 0.05).

#### Treatment group: correct trials

**Figure 4:**
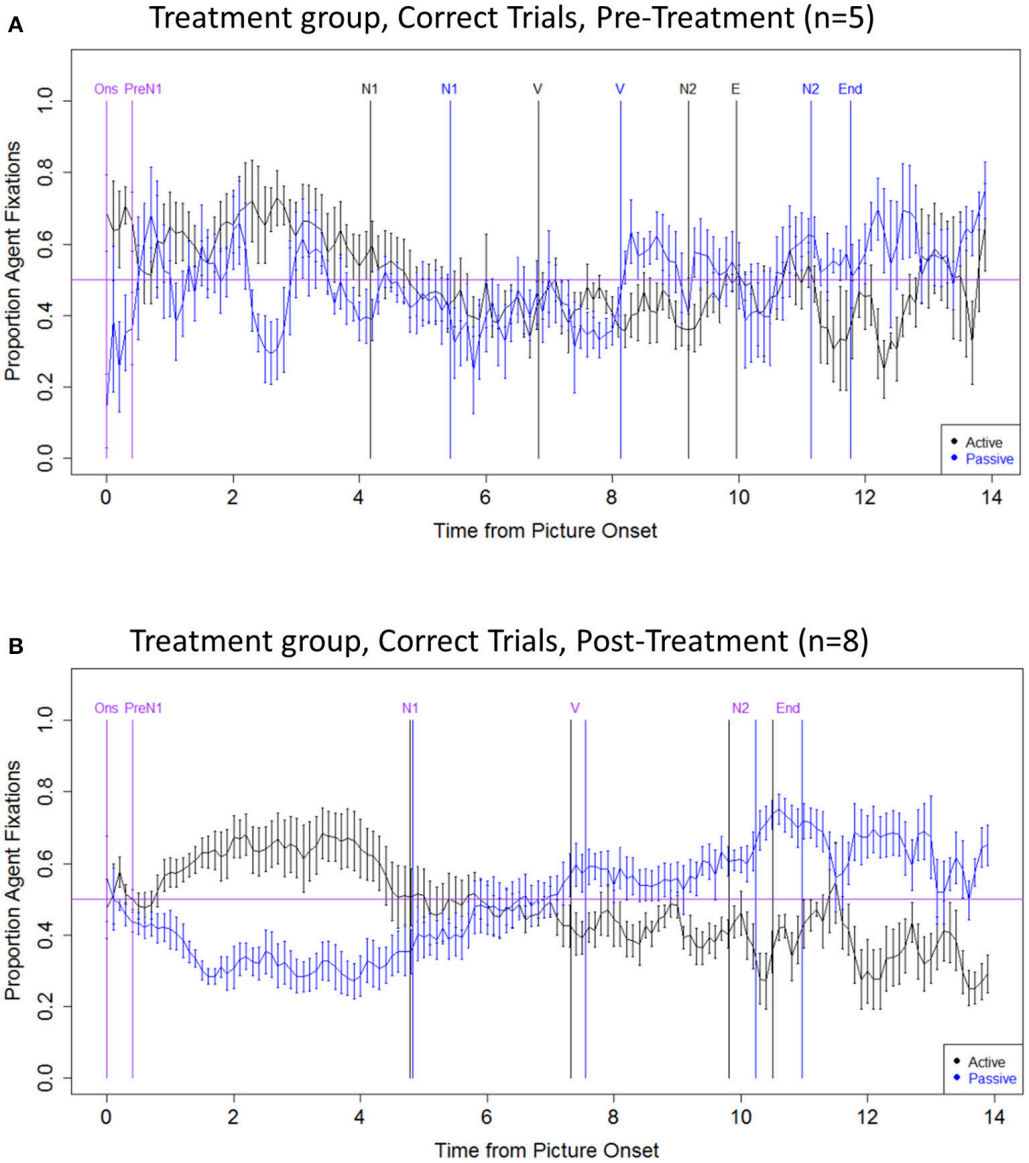
**Eye movement data: Treatment group, correct trials**. **(A)** Pre-treatment data, **(B)** Post-treatment data. The x axis indicates the time from picture onset in seconds; the y axis indicates the proportion of fixations to the Agent, out of all fixations to the Agent and Theme. Vertical lines indicate the mean onset time for each sentence region (active in black; passive in blue; labels, and onset times that are the same/similar across structures, are in purple). The horizontal line indicates at-chance fixation patterns (i.e., an equal proportion of fixations to the Agent and Theme). Ons, picture onset; PreN1, beginning of PreN1 region (400 ms); N1, onset of subject noun; V, onset of verb; N2, onset of post-verbal noun; End, trial end.

The second set of analyses examined eye movements in the correct responses produced by speakers with aphasia pre- and post-treatment (Figure 4). The data reveal changes in early eye movements (Onset region) from pre- to post-treatment. At pre-treatment, participants had more Agent advantage trials in active compared to passive sentences (*z* = −2.541, *p* < 0.05), indicating early fixations on the picture corresponding to the sentence subject. However, in the post-treatment data, there were no significant effects of sentence type in this region (*p* > 0.05), consistent with the pattern seen in unimpaired speakers. Further, visual inspection of the data indicates that the encoding of N1 and N2 was more robust and consistent in the post-treatment as compared to the pre-treatment data.

#### Natural history group: all trials

**Figure 5:**
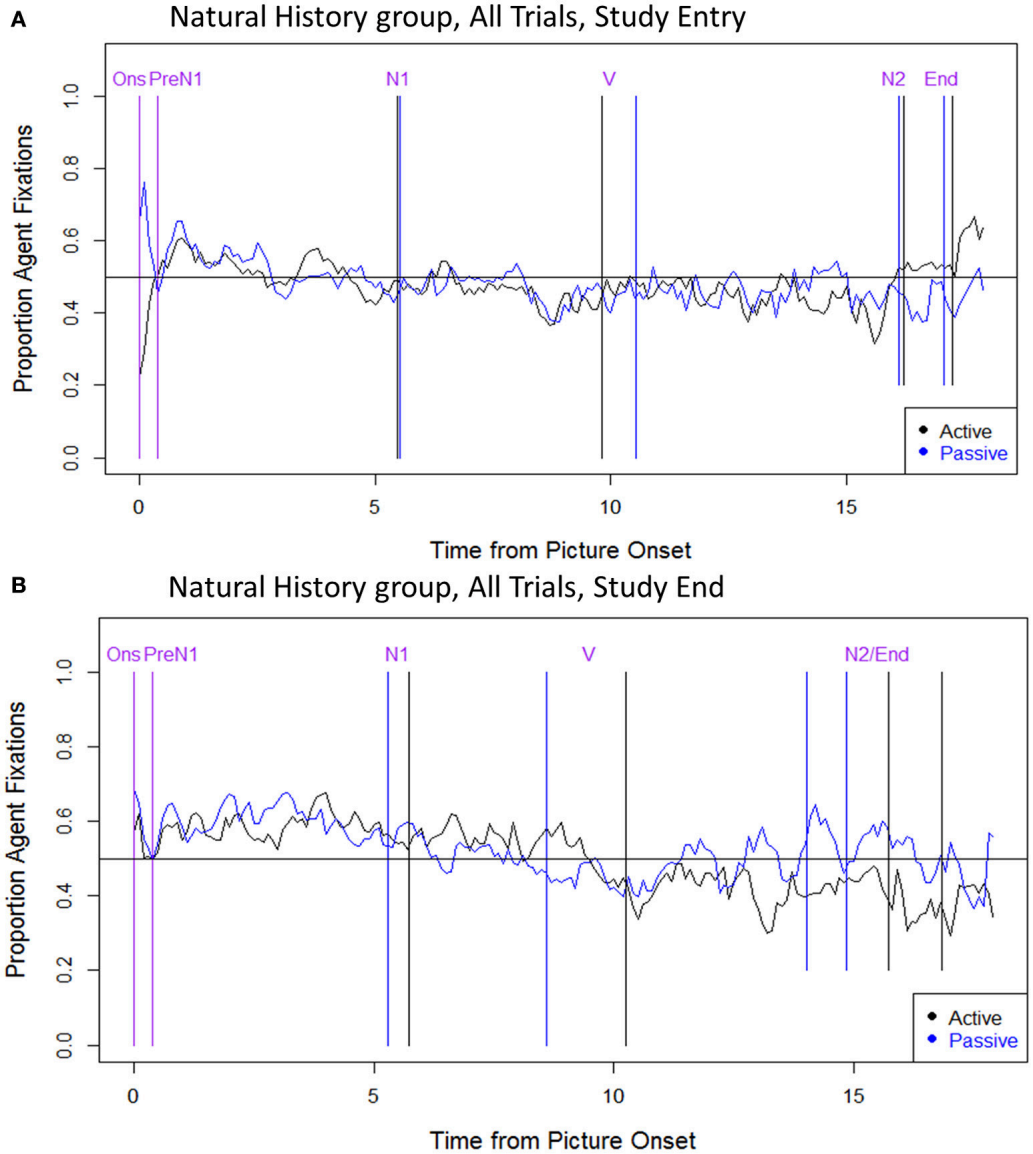
**Eye movement data: Natural History group, all trials**. **(A)** Study entry, **(B)** Study end. The x axis indicates the time from picture onset in seconds; the y axis indicates the proportion of fixations to the Agent, out of all fixations to the Agent and Theme. Vertical lines indicate the mean onset time for each sentence region (active in black; passive in blue; labels, and onset times that are the same/similar across structures, are in purple). The horizontal line indicates at-chance fixation patterns (i.e., an equal proportion of fixations to the Agent and Theme). Ons, picture onset; PreN1, beginning of PreN1 region (400 ms); N1, onset of subject noun; V, onset of verb; N2, onset of post-verbal noun; End, trial end.

The eye movement data for the Natural History group (all trials combined) are presented in Figure 5. These data revealed an abnormal pattern (with no clear differences between active and passive sentences) that was largely consistent from study entry to study end. Critically, the Natural History group did not show eye movement changes reflecting more successful encoding of the two noun phrases. Due to sparse data in this participant group we did not examine effects of test session, or perform a separate analysis of the data from correct trials.

#### Individual performance patterns: treatment effects and comparison with natural history group

**Figure 6:**
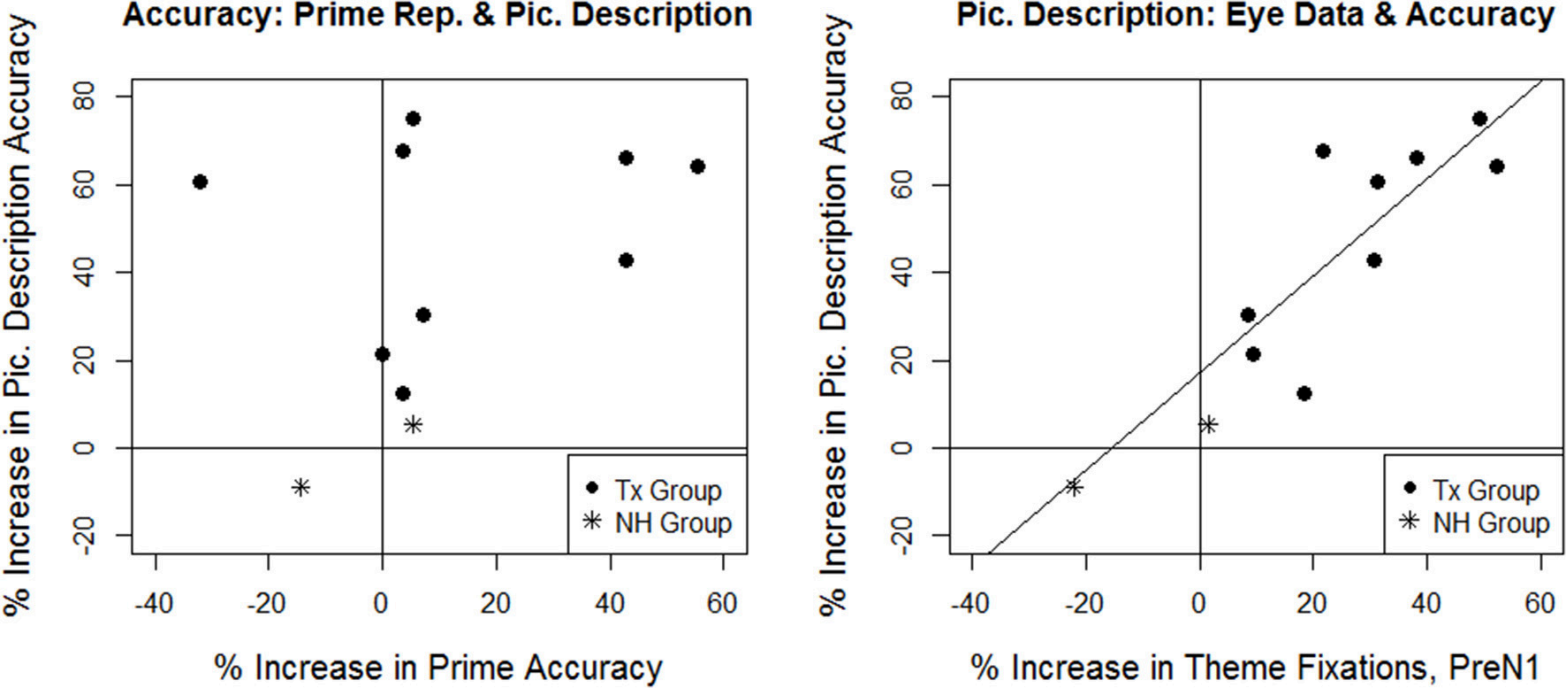
**Individual changes in passive sentence production accuracy and processing in the Treatment (Tx) group and Natural History (NH) group participants**. Points to the right of the vertical line and above the horizontal line indicate improvements in performance. The diagonal line in the second figure indicates the significant correlation between eye movements and accuracy for picture description in the Treatment group.

Figure 6 illustrates pre- to post-study changes in passive sentence production accuracy and online eye movements for participants in the Treatment and Natural History groups. In the Treatment group, seven of nine individuals showed improved prime repetition, whereas all nine individuals showed improved picture description accuracy and improvement in eye movement patterns (an increase in Theme advantage trials in the PreN1 region). There was no significant correlation between changes in prime repetition accuracy and picture description performance (accuracy and eye movements; *p*'s > 0.05). However, there was a strong positive correlation between changes in accuracy and changes in eye movements in the picture description task (Pearson *r* = 0.77, *p* < 0.05), indicating that those who showed greater gains in accuracy also showed larger changes in eye movements.

Turning to the comparison between the Natural History and Treatment groups, the Natural History participants did not differ significantly from the Treatment group with respect to changes in prime repetition accuracy (Crawford-Howell *t*-test, one-tailed, *p*'s > 0.1). However, greater improvements in picture description accuracy and online eye movements were observed in the Treatment group as compared to the Natural History participants; these effects were significant for NH01 [accuracy: *t*_(8)_: −2.41, *p* < 0.05; eye movements: *t*_(8)_: −3.05, *p* < 0.01] and approached significance for NH02 [accuracy: *t*_(8)_: −1.81, *p* = 0.054; eye movements: *t*_(8)_: −1.64, *p* < 0.070].

## Discussion

The present study examined the effects of TUF (Thompson and Shapiro, 2005) on online sentence production in speakers with aphasia. Nine participants with aphasia (Treatment group) received a 12-week course of TUF, which trained production and comprehension of complex passive sentences. A structural priming task (following Cho and Thompson, 2010) was administered twice before and twice after language treatment, with eyetracking used to monitor online sentence production. In order to distinguish treatment effects from practice effects, two individuals with aphasia (Natural History group) were tested on language probe tasks and performed the eyetracking task twice before and twice after a 12-week no-treatment period, and their performance patterns were compared to that of the Treatment group. In addition, ten unimpaired older adults performed the eyetracking task to identify normal processing routines. We tested the hypothesis that TUF would support improved thematic mapping in passive sentences, as reflected by improved sentence production accuracy. We also tested the hypothesis that TUF would result in normalization of online processing, i.e., reduced speech onset latencies and/or changes in eye movements, indicating more normal-like sentence planning. The results indicated that TUF resulted in improved sentence production as well as normalization of some, but not all, aspects of participants' online sentence production.

### Typical sentence production patterns: unimpaired older adults

The older adult speakers showed at-ceiling accuracy on repetition of the prime sentences and high accuracy (*M* = 93%) on picture description responses. For picture description, accuracy and speech onset latencies did not differ between active and passive sentences. Eye movement patterns in the unimpaired adults were consistent with structural sentence planning, with no differential fixation patterns observed across sentence types in the first 400 ms after picture onset (i.e., participants directed fixations equally to the Agent and Theme characters), in keeping with the idea that speakers used this time to conceptually encode the event and build an abstract structural frame (cf. Griffin and Bock, 2000; Konopka and Meyer, 2014). These results are consistent with the claim that structural priming paradigms facilitate the structural mode of sentence planning (Van de Velde et al., 2014). Subsequent eye movements before speech onset, in the PreN1 region, indicated encoding of N1 (i.e., the Agent in active sentence trials; the Theme in passive sentence trials), whereas eye movements after speech onset (N1 and V regions) indicated encoding of N2 (i.e., the Theme in actives and the Agent in passives), consistent with previous studies on normal language production (Griffin and Bock, 2000; Gleitman et al., 2007; Hwang and Kaiser, 2014; Konopka and Meyer, 2014; Van de Velde et al., 2014). The unimpaired speakers in the present study (*M* = 58 years) were slightly older than the participants with aphasia (*M* = 48 years). Mixed results have emerged in the literature regarding the effects of age on sentence production; many studies report subtle, if any, differences between younger and older adult speakers (Griffin and Spieler, 2006). Thus, we believe that the small mean age difference between unimpaired and aphasic participants likely had minimal impact on the pattern of results.

### Effects of language treatment: sentence production accuracy

At entry into the study, the participants with aphasia (Treatment group and Natural History group) showed poorer production of non-canonical vs. canonical sentences (Sentence Production Priming Test of the Northwestern Assessment of Verbs and Sentences, Thompson, 2011), consistent with previous studies (Faroqi-Shah and Thompson, 2003; Burchert et al., 2008; Thompson and Lee, 2009; Thompson et al., 2013a).

The participants with aphasia who received TUF showed large and consistent gains in passive sentence production, as indicated by their performance on treatment probes. At the group level, passive sentence production accuracy increased from a mean of 12% correct pre-treatment to 81% correct post-treatment, and all participants showed improvement at the individual level. The magnitude of this training effect is comparable to what has been observed in previous TUF studies. In a meta-analysis, Dickey and Yoo (2010) found that TUF resulted in pre- to post-treatment gains of approximately 77% for trained sentences. The results of the present study are largely in line with these findings. Importantly, these gains were specific to the Treatment group, as the Natural History participants showed stable sentence production accuracy from study entry to study end. Furthermore, the Treatment group showed stable performance from pre- to post-treatment on a set of control tasks, which measured language processes unrelated to those targeted by TUF, suggesting that improvement was specific to trained language domains (i.e., verb and sentence processing).

Similarly, improved production of passive sentences was observed for participants in the Treatment group in the prime repetition and picture description components of the structural priming (eyetracking) task. In contrast, the performance of the Natural History participants on both components of the task was stable, with minimal change from study entry to study end. However, for the prime repetition component of the task, the changes in performance for the Treatment and Natural History groups did not reliably differ. Therefore, we cannot rule out the possibility that other factors contributed to these improvements (e.g., practice effects). Moving to the picture description component of the task, accuracy of passive sentences improved substantially for all nine Treatment group participants (pre-treatment *M* = 14%; post-treatment *M* = 63%), consistent with the behavioral probe results. These improvements in performance were significantly greater than changes in performance observed in the Natural History group, suggesting that they are language treatment effects. Notably, the picture description task was designed with the goal of isolating thematic mapping—i.e., the use of thematic information to correctly encode the grammatical subject and post-verbal noun phrase, given the primed verb and syntactic structure, whereas the prime repetition component of the task did not require thematic mapping. The primary focus of TUF also is to train thematic mapping, and thus participants' improvement on picture description reflects not only learning, but also generalization to untrained material, given that there was no overlap between the trained sentences and the sentences included in the structural priming task.

In addition, the results indicated treatment-related changes in the frequency of different types of errors in the picture description task. Treatment group participants evinced a significant decrease in the rate of Incomplete/No Response/Multiple Type errors for passive sentences (from 44 to 13% of all trials) and a numeric decrease for actives (from 28 to 15% of all trials), indicating a substantial reduction in the rate of the most severe sentence production errors. Additionally, the rate of Correct Unprimed Structure Errors (i.e., production of a grammatically and thematically correct active following an passive prime, or vice versa) decreased with treatment for passive primes (from 18 to 5% of all trials). In contrast, error patterns remained largely stable from study entry to study end in the Natural History group.

### Effects of language treatment: online processes

Online processing in the picture description task also changed significantly following language treatment. When all trials (correct and incorrect) were combined, there was no difference in pre-treatment eye movements between the active and passive prime conditions—an abnormal pattern, indicating faulty encoding of the Agent and Theme noun phrases. However, following treatment, participants fixated the Agent significantly more often in actives than passives in the PreN1 region, indicating improved encoding of the sentence subject. Eye movement patterns in the V and N2 regions also indicated improved encoding of N2 (i.e., more time spent fixating the Agent in passives than in actives). The changes in eye movement patterns were driven by passive sentences (i.e., a post-treatment increase in Theme fixations in the PreN1 and N1 regions, and Agent fixations in the V and N2 regions). These findings confirm our hypothesis that improved sentence production would be associated with more normal-like eye movement patterns, indicating sequential encoding of the correct noun phrase in the N1 region (before speech onset) and in the N2 region (after speech onset). Notably, we observed a relationship between changes in sentence production accuracy and eye movements on the picture description task. Improved accuracy for passives was strongly correlated (*r* = 0.77) with an increase in Theme fixations in the PreN1 region, indicating that increased accuracy was supported by more reliable encoding of the grammatical subject. The Natural History participants also showed abnormal eye movement patterns at study entry, with no clear differences observed between active and passive sentences. However, unlike the Treatment group, these patterns were also observed at study end, suggesting that eye movement changes in the Treatment group resulted from treatment.

Treatment-related eye movement changes were also observed when the analyses were limited to trials in which participants produced a correct picture description response. At pre-treatment, correct trials showed abnormal eye movements. In the first 400 ms after picture onset, the aphasic speakers fixated the Agent in active sentences and the Theme in passive sentences, whereas unimpaired controls showed no differential fixation patterns in this early period. In healthy speakers, differential fixation patterns have been argued to reflect word-by-word planning i.e., selection of the first-fixated character as the grammatical subject (e.g., Gleitman et al., 2007; Kuchinsky and Bock, 2010; Van de Velde et al., 2014), which is dispreferred in the context of explicit structural priming paradigms and, in aphasic speakers word-by-word planning is little used (Lee and Thompson, 2011a,b; Lee et al., 2015). After language treatment, however, the aphasic speakers showed no differential fixation patterns in the first 400 ms after picture onset, as in our healthy control participants. Further, post-treatment trials showed evidence of successful encoding of the correct noun phrase in the N1 and N2 regions, which was not evident in the pre-treatment data. These findings suggest that the pre-treatment eye movement patterns for correct trials did not reflect word-by-word planning, but rather reflected aberrant structural planning. Specifically, we suggest that in these trials, the syntactic structure was primed successfully but thematic mapping did not take place normally. Instead, the most accessible or salient character was selected as the grammatical subject—i.e., the one that was fixated just after picture presentation. This interpretation is consistent with that of Cho and Thompson (2010), who found intact priming of morphosyntactic structure with a high rate of thematic mapping (role-reversal) errors. These findings suggest that TUF resulted in successful training of structural planning. Interestingly, similar changes in eye movement patterns were observed in our previous study examining the effects of TUF on online sentence comprehension (Mack and Thompson, in press). In that study, pre-treatment correct trials showed evidence of a non-grammatical response bias—that is, a tendency to select the picture fixated early in the sentence—that disappeared after treatment, and was replaced by a more normal-like pattern, reflecting improved thematic mapping.

In contrast, two aspects of online sentence production remained atypical following treatment. First, the participants with aphasia exhibited protracted speech onset latencies, which were more than twice as long (conditional means ~4–5 s) as those of unimpaired speakers (conditional means ~2 s) and remained unchanged with treatment. Second, some aspects of sentence planning appeared to differ between unimpaired speakers and speakers with aphasia, even in the post-treatment data. The eye movements of the unimpaired speakers indicated rapid and highly incremental processing. For example, shortly before speech onset (i.e., the production of N1), participants began to shift their gaze toward the picture corresponding to N2 (the Agent in passives and the Theme in actives), indicating temporal overlap in the encoding of the two noun phrases. In contrast, the post-treatment eye movements of speakers with aphasia indicated encoding of N2 after production of the first noun phrase was completed. This pattern may indicate reduced incrementality in speakers with agrammatism, even after receiving language treatment. This may relate to a preference of the aphasic production system to avoid simultaneous encoding of two noun phrases, which may be costly and thus avoided (see related discussion in Lee et al., 2015). TUF in its current form does not emphasize sentence production speed, or incremental processing, and thus it is perhaps unsurprising that these aspects of sentence production did not change with treatment. However, in future work, it could be fruitful to develop new modules of TUF aimed at training rapid, incremental sentence production, and to evaluate their effects on recovery of sentence production.

Notably, accuracy, eye movements, and speech durations were stable across the two pre-treatment test sessions and two post-treatment sessions, for both passive and active sentences. Crucially, the stable performance observed within each phase (and across measures) supports the idea that changes from pre- to post-treatment in the picture description task reflect true treatment effects, rather than practice effects. Consistent with previous research, these findings suggest that eyetracking can provide a stable measure of language recovery in individuals with aphasia (Mack et al., 2016; Mack and Thompson, in press).

## Conclusion

To our knowledge, the present study provides the first demonstration of how real-time sentence production changes in response to language treatment (TUF) in aphasia. These findings provided insight into the mechanisms of TUF, and also revealed aspects of sentence production that improved with treatment as well as those that remained unchanged, which could be addressed in novel treatment protocols. In future research, it will be informative to examine how different approaches to sentence production treatment affect the sentence processing system, as well how (and if) changes in online processing relate to neural aspects of the recovery of sentence processing (Wierenga et al., 2006; Thompson et al., 2010, 2013b). By providing a window into how language is processed, online methods such as eyetracking have the potential to advance our understanding of the neurocognitive mechanisms of language recovery.

## Author contributions

CT and JM designed the study and oversaw all aspects of data collection, analysis and interpretation, and manuscript writing and revision. MN contributed to data collection, analysis, and interpretation of results.

### Conflict of interest statement

The authors declare that the research was conducted in the absence of any commercial or financial relationships that could be construed as a potential conflict of interest.

## Footnotes

^1^This study is part of a larger project examining the effects of treatment on several measures of neurocognitive processing, including measures of brain structure and function as well as measures of language and cognitive processing.

^2^One individual (A09/NH02) was first enrolled in the Natural History group and then (after a 12 week interval) in the Treatment group.

^3^Participants A01-A09 were also included in our previous study examining the effects of the current TUF protocol on online sentence comprehension (Mack and Thompson, in press). Participant A10 from the Mack and Thompson comprehension study was unable to perform the eyetracking production task at pre-treatment and was thus excluded from the present study.

^4^A01 received training for 6 weeks.

^5^These sentences were selected to test hypotheses about patterns of learning and generalization.

^6^These measures were unavailable for A01.

^7^A01 only took part in a single pre-treatment eyetracking session.

^8^In the case of non-convergence, we first reduced the random effects of Item, and then (if necessary) the random effects of Participant. In a few cases, random effects for Participant were eliminated due to near-zero estimation (β < 0.001).

^9^Simple effects were tested by replacing the global interaction with two simple effects (e.g., replacing the Phase ^*^ Structure interaction with simple effects of Phase for each Structure), keeping other aspects of the model structure constant.

^10^The *p-*values for Phase, Sentence Type, and Phase ^*^ Sentence Type, for all error types, were entered into a single FDR correction.

^11^A logarithmic transformation was used for the data from the Treatment group, whereas a reciprocal transformation was used for the data from unimpaired controls. For the Treatment group, two speech onset latency values greater than log = 10.5 were removed from the analysis of all trials combined (0.1% of data); one speech onset latency value greater than log = 9.5 was removed from the pre-treatment correct responses (0.6% of data). No outliers were removed from other data sets.

^12^For the analysis of eye movements from all trials (regardless of response accuracy), the *p*-values for Phase, Sentence Type, and Phase ^*^ Sentence Type, for all sentence regions, were entered into a single FDR correction. For the analyses of Session effects, the p-values for Session, Sentence Type, and Session ^*^ Sentence Type, for all sentence regions, were entered into FDR corrections, separately for the pre-treatment and post-treatment data. For the analyses of eye movements in correct trials, the p-values for Sentence Type, for all sentence regions, were entered into FDR corrections, separately for the pre-treatment and post-treatment data.
